# Supported employment for people with severe mental illness: a pilot study of an Italian social enterprise with a special ingredient

**DOI:** 10.1186/s12888-022-03881-8

**Published:** 2022-04-26

**Authors:** Alessandra Martinelli, Chiara Bonetto, Federica Bonora, Doriana Cristofalo, Helen Killaspy, Mirella Ruggeri

**Affiliations:** 1 Department of Neurosciences, Biomedicine and Movement Sciences, University of Verona, Piazzale L.A. Scuro 10, 37134 Verona, Italy; 2 Unit of Clinical Psychiatry, IRCCS Istituto Centro San Giovanni Di Dio Fatebenefratelli, Via Pilastroni, 4, 25125 Brescia, BS Italy; 3 Division of Psychiatry, University College London, London, UK; 4 Section of Psychiatry, Verona Hospital Trust, AOUI, Verona, Italy

**Keywords:** Supported employment, Social cooperative, Social enterprise, Severe mental illness

## Abstract

**Background:**

People with mental disorders are far more likely to be unemployed than the general population. Two internationally recognized, evidence-based models of interventions for employment for people with severe mental health problems are Individual Placement Support and the Clubhouse. In Italy, a common model is the ‘social enterprise’ (SE), which is a programme run by non-profit organisations that help individuals with disabilities to be employed. Despite SEs spread and relevance in Italy, there are no studies about Italian samples. This paper reports on a pilot evaluation of psychosocial and work outcomes of a SE based in Verona, Italy. The study aims to investigate if people with SMI involved in SE job placements may achieve personal recovery and better outcomes over time, and in comparison with a comparable group of users.

**Methods:**

This is a pilot descriptive study with three components. A longitudinal design that comprised a functioning description of 33 SE members with a psychiatric disability in two time-points (when they joined the SE—on average 5 years before the study recruitment, and at the study recruitment—year 2018); and a repeated collection of job details of the 33 members in three time points: 2 years before the recruitment,—year 2016; 1 year before the recruitment – year 2017; and at the recruitment—year 2018. An assessment at the recruitment time—year 2018, of SE users’ satisfaction with the job placement, symptoms, functioning, and quality of life (QoL). A cross-sectional study that compared the 33 SE members at the recruitment time—year 2018, with a matched group of people with the following criteria: living in local supported accommodations, being unemployed and not SE members. The two groups were compared on ratings of psychopathology, functioning, and QoL. Descriptive analyses were done.

**Results:**

At the recruitment time – year 2018, all SE participants showed a significant better functioning (*p* < 0.001) than when they joined the SE—when they had been employed for an average of 5 years. In comparison to the matched group, SE members had significantly better functioning (*p* = 0.001), psychopathology (*p* = 0.007), and QoL (*p* = 0.034). According to their SE membership status, participants comprised trainees (21.2%) and employee members (78.8%). Trainees compared to employees had lower autonomies, functioning, QoL and more severe psychopathology. Over the two years prior to study recruitment, trainees showed stable poor autonomies, while employee members showed a variation from average autonomies in the 2 years before the recruitment time – year 2016, to good ones at the recruitment time – year 2018. Over the two years, all SE members set increasing numbers of objectives in all three domains. All SE participants reported high levels of satisfaction with all aspects of the job placement.

**Conclusions:**

SE that provides tailored support to assist people to gain employment skills may be an effective component in helping recovery from SMI.

**Supplementary Information:**

The online version contains supplementary material available at 10.1186/s12888-022-03881-8.

## Introduction

Competitive employment is one of the principal signifiers of normal adulthood. However, people with severe mental illness (SMI), with difficulties in functioning in daily life, long-term illness and including all forms of mental disorders [1], are more likely to be unemployed than the general population. It is estimated that only 10% to 20% of people with SMI in Europe have a job [2, 3], and they are twice as likely to become unemployed after the onset of the disorder [4]. Nevertheless, the achievement of competitive employment is an important goal for people with SMI [5, 6].

Employment has been shown to be associated with positive outcomes for people with mental disorders and for society, reducing the costs associated with mental illnesses [7, 8]. It impacts positively on people’s mental health and leads to improvement in motivation, self-confidence, perceived quality of life, and social support [6, 9–11]. Thus, different interventions aimed at employment of people with SMI have been developed across Western countries [12]. Evidence is strongest for the effectiveness of the *Individual Placement Support* (IPS) model, which aims to support people to find and sustain competitive employment directly rather than requiring any pre-employment vocational rehabilitation [13–15]. However, the *Clubhouse* model, is also popular internationally, providing prevocational training in a recovery oriented context where people join as members, co-run the project and develop social and work related skills [16].

In Italy, since the deinstitutionalization process started in the 1960s, and after the radical reform of 1978 [17], programs aimed at employment have been considered good practice for mental health care. A quota of working places for citizens with a disability by public and private employers was established by Italian law 68/1999 [13]. Social cooperatives or ‘co-ops’ have taken on this function widely across the country [10]. Social co-ops are not-for-profit providers/organizations of various services and industries that operate with a collaborative, and membership model. They are thousands on the Italian soil, and can be distinguished in two types: Type A and Type B.

Type A social cooperative may act as providers of supported accommodation or home care support for people with a disability.

Type B co-ops offer employment to people with social disability, including people with mental or physical problems [11, 13]. These co-ops develop programmes that help individuals with disabilities to be employed in non-competitive market, and since Law 106/2016 and Law 117/2017, in the competitive employment setting too, so Type B co-ops can also be called ‘social enterprise’ (SE).

In detail, SEs are semi-commercial business which offer paid employment at competitive rates for people with social disability and with difficulty in entering into the normal labour market [18–21]. In an integrated working environment, groups of people with disability are trained and supervised by other workers with or without a disability [22].

SEs can establish agreements both with public services (supply of social, health, and educational services and goods) as long as they aim to create job opportunities for people with a disability, and with private companies (e.g. for the assembly and packaging of goods, or providing cleaning). Every Italian Region regulates the work of SE, predisposing pathways and a set of mandatory work documents [23]. These documents are all compiled by cooperative employees without a disability in relation to employees with a disability and comprise job placement projects with assigned tasks, work contracts, performance evaluations, and specific objectives to achieve.

To be registered as a SE at least 30% of employees must have a disability. The most recent available data indicate that Italian SEs have an average of 54.5% members with a social disability [11, 24–26].

The flexibility of the SE model [27] has enabled employment of thousands of Italian people with severe mental health problems, albeit often in low or unskilled positions [10, 21].

SE is worldwide studied because this model is able to support and place in the work market people with long-term mental illness and high needs [23, 28], help them in maintaining a standard job (where working time, health and safety requirements and responsibilities are regulated [29]) for between 2 and 6 years [19, 28], increase their recovery [19, 30, 31], and reduce perceived stigma [27]. However, despite the notable relevance of SE in Italy, there are neither descriptive nor experimental studies about Italian samples [12].

This study aimed to evaluate if people with SMI involved in SE job placement:

-
  - presented an improvement in psychosocial (e.g. functioning, symptoms, quality of life) and work outcomes over time such as job maintenance, work skills and autonomies, and job in the competitive market;
-
  - presented an improvement in psychosocial outcomes in comparison with a matched group of people with SMI without an employment;
-
  - implement their skills to achieve personal recovery including how to live as fulfilling a life as possible despite psychiatric disability and cover all the major roles of adulthood [32], and empowerment.

To pursue the study aims, we developed a descriptive pilot study on a SE based in Verona, Italy, for people with severe mental problems that, inspired by recovery principles, includes components of both IPS and the Clubhouse approach.

### The studied social cooperative and the characteristics of the employment of the users

The social cooperative evaluated in this project was founded in 2006 and has always collaborated closely with the Verona Mental Health Department. It is both a Type A and Type B cooperative. Its Type A component involves the provision of supported accommodation for people with mental problems, while its Type B function comprises personalized job placements for people with mental disorders, which aim to facilitate social inclusion within a strong recovery orientated ethos [33].

The cooperative has developed a number of ‘place and support’ employment programs that mirror the characteristics of posts in mainstream employment. These placements are provided within five main work areas: laundry, green area/garden maintenance, forecourt surveillance, and restaurant and hotel services, all within the Lake Garda area. The laundry service is provided to local residential care homes and hotels, the garden maintenance service is provided to private houses and apartment blocks, and the forecourt surveillance service is provided to some of the larger local supermarkets. The SE’s popular restaurant provides employees with opportunities to gain skills in all aspects of the restaurant business (kitchen, front of house, reservations, waiting on tables etc.). The restaurant is supplied with local fruit and vegetables produced through another arm of the cooperative, and it also supplies breakfasts to nearby hotels. The SE usually has around 50 employees, of whom about three-quarters have a disability related to mental health problems, and one quarter are mental health professionals. Potential employees with mental health problems are referred to the cooperative by the local mental health services, or from other private or public bodies that deal with job placement, internship, or vocational training. On starting, they initially complete a pre-vocational work training lasting 3, 6 or 12 months in a specific role, agreed after an initial assessment and taking account of any previous work/education experience and preferences. During the training, a mental health professional member of the cooperative and the cooperative supervisor evaluate the trainee’s performance to decide if s/he can become an employee member, or whether they need to extend the training or stop/interrupt the job placement process.

According to regional guidelines, performance is measured on the basis of the achieved level of autonomy (understood as 'a person's ability to provide for his or her own needs') in personal (health, mental health, and self-management), social (relationship with others and social behavior) and work (basic and more specialist) skills areas. When the trainee becomes an employee member of the cooperative, they sign a fixed term (3, 6, or 12 months) contract.

The entry salary for each employee member is set according to a specific level that is commensurate to their skills and responsibilities (from level A1 corresponding to ‘cleaning, custody, generic duties’ to level F2 ‘workers with functions of great responsibility for the development and strategies of the cooperative’) [34]. Employees with social disabilities usually start at the A1 level. The employee member is also assigned an ‘entry salary range’ somewhere between 50 and 100% according to their autonomy to carry out the assigned task.

After the initial contract period, further contracts and salaries are agreed based on the person’s performance/progress on their personal, social and work autonomies, which are reviewed and adjusted as needed every 3, 6 or 12 months. Employees with a permanent contract have a review of their performance every year to verify the maintenance of their autonomies, and, if necessary, to re-evaluate tasks and job hours.

## Methods

### Study design

This study was a descriptive pilot one. The study design included three components:

i) a longitudinal study that comprised:
  an evaluation of SE users’ functioning in two time-points, the first when they joined the SE (T0) (on average 5 years before the study recruitment) and the second at the recruitment time (T1) (year 2018);
  a repeated collection of job details (personal, social and work autonomies and related objectives of SE members) 2 years before the recruitment (T3) (year 2016); 1 year before the recruitment time (T2) (year 2017) and at the recruitment time (T1) (year 2018) (no further retrospective data were available);
ii) an assessment at the recruitment time (T1) (year 2018) of SE users’ satisfaction with the job placement, symptoms, functioning, and quality of life (QoL);
iii) a cross-sectional study comparing SE members with SMI at the recruitment time (T1) (year 2018) and a group of people with SMI unemployed matched by primary psychiatric diagnosis and years of contact with mental health services aiming to compare symptoms, functioning and quality of life (QoL).

### Participants’ recruitment

Employees of the SE with a social disability were included in the study if they fulfilled the following eligibility criteria*:*

i) primary diagnosis of a severe psychiatric disorder (ICD-10) including psychoses (F20-F29), affective disorders (F30-39), anxiety disorders (F40-F48), personality disorders (F60-F69) or other long term mental health problems (F80-F99);
ii) age between 18 and 65 (working age);
iii) minimum 4 months of work in the cooperative on the 15^en^ December 2018, corresponding to the recruitment time (T1); iv) able to give informed consent to participate.

Exclusion criteria: *i)* diagnosis of moderate or severe mental retardation (ICD-10: F71-F79); *ii)* primary diagnosis of mental disorders due to physiological, organic, physical, and/or psychoactive substances (ICD-10: F00-F19, F50-F59).

The matched comparison group was identified from the pool of residents of local mental health supported accommodation services who were not employed in any work activities in the SE or elsewhere but had significant mental health problems as those of the SE.

### Assessments of the supported employment characteristics

The data collated on study participants from the social co-op records and interviews by researchers were: previous work experience; work role in the SE (trainees or employee membership); months of work in the SE; assigned task in the SE; personal, social and work autonomies and objectives in the two years prior to and at study recruitment.

Researchers rated each participant’s personal, social and work autonomy using a 5-point Likert scale (from 1 = poor autonomy to 5 = excellent autonomy) and categorized their placement objectives according to the same three subgroups: personal objectives 6 sub-typologies); social objectives (6 sub-typologies); and work objectives (8 sub-typologies). Researchers analysed the autonomies and objectives proposed by MH professionals in December 2018 (recruitment time = T1), then investigated retrospectively the autonomies and objectives given in December 2017 (1 year before the recruitment time = T2) and further analysed retrospectively those proposed in December 2016 (2 years before the recruitment time = T3).

Main objectives proposed to achieve were: *i)* mental health management, *ii)* moving to an independent living, *iii)* minimum skills necessary to maintain a job and to manage daily activities (e.g. keeping personal hygiene and order, being on time, travelling independently from to the workplace), *iv)* the participation to social activities proposed by the co-op to increase social skills, social network and assertiveness *v)* the increase of work responsibilities, reduction of supervision, and growth of personal initiative, *vi)* developing or strengthening work skills, *vii)* the reduction of workload or hours/week and work on motivation. Objectives related to the observation of the autonomies in each area were objectives proposed for trainees so to evaluate if they could become an employee member, or not.

Data on SE members’ satisfaction with their job placement were also collated from self-report satisfaction questionnaires [at the recruitment time = T1] that used a 7-point Likert scale (rated from 1 = the worst possible job to 7 = the best possible job).

### Assessment of users

Standardised instruments were completed to assess co-op members’ psychosocial functioning, psychopathology, subjective quality of life, and satisfaction with services, as follows:

-
  - Psychosocial functioning was evaluated by researchers with discussion with mental health professional who knew the person well., and with reference to case records, using the *Global Assessment of Functioning Scale* (GAF), which produces an overall rating from 0 to 100 with higher scores denoting higher functioning [35]. Particularly, for SE members, two independent researchers collected data from case records and professionals to evaluate retrospectively their functioning when they joined the SE (T0).
-
  - The severity of psychopathology was assessed by the researchers through face-to-face interviews with participants and discussion with mental health professional who knew the person well. The Italian version of the *Brief Psychiatric Rating Scale, expanded version* (BPRS) [36], which comprises 24 items rated on a seven-point Likert scale (from 1 = no symptoms to 7 = extremely severe symptoms) scale, producing a total mean score from 1 to 7 was used. Sub-scales also provide ratings on 5 areas (anxiety-depression, negative symptoms, positive symptoms, mania/excitement, cognition) [37].
-
  - Subjective quality of life (QoL) was assessed using the *Manchester Short Assessment of Quality of Life* (MANSA) that, completed with the assistance of the researchers if needed, rates each of 11 life domains (e.g. living situation, employment situation, relationships, physical and mental health) on a seven-point Likert scale (from 1 = not at all satisfied to 7 = extremely satisfied) producing a total mean score from 1 to 7. Sub-scales also provide ratings on 2 dimension (living, health) [38].
-
  - Socio-demographic, service use and clinical data were obtained from professionals and using the Verona Mental Health Department database and South-Verona Psychiatric Case Register-PCR [39];

### Statistical analysis

The data were analysed using SPSS statistical software for analysis for Windows. Descriptive statistics (frequencies and percentages for categorical variables, mean values ​​and standard deviations for continuous variables) were first generated.

SE users were described according to their working status: trainer or employee member). No comparisons were performed between these two subgroups because of too small and unbalanced sample sizes.

Comparisons between the SE members group and the comparison group were made using the Pearson Chi-square test for categorical variables and the t test for independent samples for continuous variables. The paired sample t-test was performed for longitudinal continuous data.

All *p*-values were two-tailed with an accepted significance level of at least 0.05. No corrections for multiple tests were applied because of the descriptive purpose of the research.

## Results

### Participants enrollment

**Fig. 1:**
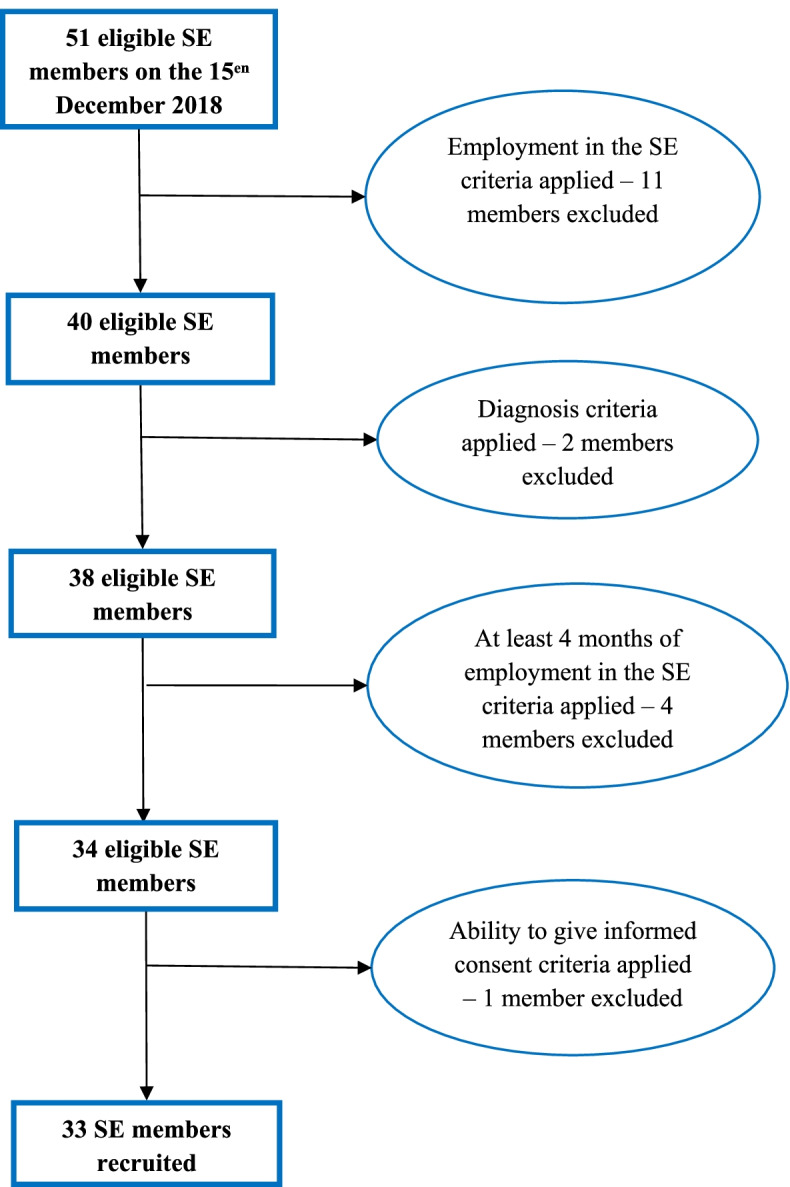
The flow-chart of the recruitment of SE members with a social disability

Fifty-one members were identified as being in contact with the SE during December 2018. Eleven were excluded because they were not currently employed in the co-op, two because they did not meet diagnostic criteria, four because they had worked in the SE for less than four months, and one was unable to give informed consent to participate due to lack of capacity. Therefore, a total of 33 (64.0%) SE members were recruited for the study (see Fig. 1).

### Job characteristics of SE participants

As shown in Table 1, SE participants were mostly engaged in tasks related to the ‘Restaurant and hotel’ sector (48.5%). They were engaged in the SE activities (T0) for about 66 months (equal to 5.5 years, SD = 5.1) ranging from 4 to 199 months, with a working range from 2 to 36 h per week.

**Table 1:** Description of job characteristics, autonomies, functioning (GAF), symptoms (BPRS), and quality of life (MANSA) of SE participants (trainees and employee members) at the recruitment time (T1 – year 2018)

	**Trainees** ***N* = 7 (21.2%)**	**Employees** ***N* = 26 (78.8%)**	**Total** ***N* = 33 (100%)**
**Job characteristics**			
*Work area, N (%)*			
Laundry	1 (14.3%)	10 (38.5%)	11 (33.0%)
Green areas maintenance	1 (14.3%)	2 (7.7%)	3 (9.1%)
Service forecourt surveillance	0 (0%)	3 (11.5%)	3 (9.1%)
Restaurant and hotel	5 (71.4%)	11 (42.3%)	16 (48.5%)
*Mean (SD)[range] number of months working in the cooperative (from T0 to T1)*	22.3 (29.5) [4–89]	77.9 (63.0) [4–199]	66.2 (61.6) [4–199]
*Mean (SD)[range] working hours per week*	8.6 (6.3) [2-22]	18.4 (8.6) [6-36]	16.3 (16.3) [2-36]
*Contract, N (%)*	0 (0%)	26 (100%)	26 (78.8%)
Time contract part-time	-	26 (100%)	26 (100%)
Work contract permanent	-	16 (61.5%)	16 (61.5%)
temporary	-	10 (39.5%)	10 (39.5%)
Entry salary 50%-80%	-	17 (65.4%)	17 (65.4%)
80%-100%	-	9 (34.6%)	9 (34.6%)
*Previous job experiences, N (%)*	6 (85.7%)	23 (88.5%)	29 (87.9%)
Manufacturing-worker sector	3 (42.9%)	12 (46.2%)	14 (48.3%)
Catering sector	2 (28.6%)	4 (15.4%)	6 (20.7%)
Other	1 (14.3%)	1 (3.0%)	2 (6.9%)
*Previous employment status, N (%)*			
Permanent employment	0 (0%)	1 (4.2%)	1 (3.0%)
Non-standard employement	2 (28.6%)	9 (34.6%)	11 (33.3%)
Claiming an invalidity pension	3 (42.9%)	9 (34.6%)	12 (36.4%)
Unemployed	2 (28.6%)	5 (19.2%)	7 (21.2%)
Unknown	0 (0%)	2 (7.7%)	2 (6.1%)
**Autonomies, Mean (SD) (1 = very poor; 5 = excellent)**			
*Personal autonomy*	2.0 (0.0)	3.9 (0.9)	3.4 (1.1)
*Social autonomy*	1.8 (0.5)	3.7 (0.8)	3.4 (1.1)
*Work autonomy*	2.0 (0.0)	3.6 (1.0)	3.3 (1.1)
* Total*	1.9 (0.1)	3.7 (0.8)	3.4 (1.0)
	(5)		(31)
**Functioning—Mean (SD) GAF score (max = 100)**	41.9 (15.3)	65.5 (7.8)	60.5 (13.7)
**Symptoms—Mean (SD) BPRS score**			
**(1 = no symptom; 7 = very severe symptom)**			
*Depression/anxiety*	2.2 (0.4)	1.9 (0.6)	1.8 (0.6)
*Negative symptoms*	1.8 (0.8)	1.7 (0.6)	1.6 (0.6)
*Positive symptoms*	1.9 (0.9)	1.3 (0.3)	1.5 (0.4)
*Manic excitement*	1.5 (0.9)	1.3 (0.4)	1.5 (0.7)
*Cognitive symptoms*	1.5 (0.7)	1.2 (0.3)	1.3 (0.5)
*Total*	1.9 (0.4)	1.5 (0.2)	1.6 (0.3)
**Quality of life—Mean (SD) MANSA main items**			
**(1 = not at all satisfied; 7 = extremely satisfied)**			
Living	4.0 (1.0)	5.3 (1.5)	5.0 (1.5)
Health	3.5 (1.6)	4.8 (1.5)	4.5 (1.6)
Total	4.3 (1.1)	5.0 (1.1)	4.9 (1.1)

Out of 33, twenty-nine (87.9%) SE participants had previous job experience: 14 (45.5%) in the manufacturing-worker sector, 6 (20.7%) in the catering sector and 2 (6.9%) in other sectors. Only few have been in standard employment [29]: only one (3.0%) participant was in permanent employment, and 11 (33.3%) were non-standard employees, 2 of whom were working in seasonal employment and 2 were close to the expiry of the contract. Twelve (36.4%) were claiming an invalidity pension, and 7 (21.2%) were unemployed.

On a 5-points Likert scale, SE participants showed average-good personal, social, and work autonomy (3.4, SD = 1.0), markedly difficulties in only one area among self-care, social and work of functioning (mean GAF score 60.5, SD = 13.7), mild psychopathological symptoms (mean BPRS score 1.6, SD = 0.3), particularly severe in depression/anxiety area (1.8, SD = 0.6), and a satisfied QoL (mean MANSA score 4.9, SD = 1.1).

Table 1 shows that, according to their SE membership status, participants comprised 7 trainees (21.2%) and 26 employee members (78.8%) with social disability.

The subgroup of trainees has joined the SE by few months (22.3, SD = 29.5, ranging from 4 to 89 months), while the group of employee members have joined the SE by many months, (77.9, SD = 63.0, ranging from 4 to 199 months).

Trainees had fewer hours per week of work with the SE compared to employee members (respectively a mean of 8.6, SD = 6.3, and 18.4, SD = 8.6 h).

It is noteworthy that 26 employee members had part-time contracts and 16 had a permanent contract (61.5%). Three-quarters (65.4%) had an entry salary between 50 and 80% of the maximum, while 34.6%. between 80 and 100%

Trainees compared to employees had lower autonomies (1.9, SD = 0.1 vs 3.7, SD = 0.8), lower functioning (mean GAF score 41.9, SD = 15.3 vs 65.5, SD = 7.8), more severe mental health symptoms (mean BPRS score 1.9, SD = 0.4 vs 1.5, SD = 0.2), especially in terms of positive symptoms (1.9, SD = 0.4 vs 1.3, SD = 0.3), and lower QoL (mean MANSA score 4.3, SD = 1.1 vs 5.0, SD = 1.1).

As expected, over the two years prior to study recruitment (see Supplementary Table 1), trainees and employees presented different ratings of personal, social and work autonomy (measured on a 1 to 5 Likert scale). Trainees throughout the two years showed stable poor personal, social, and work autonomy (2.0, SD = 0.0), while employee members showed a variation in autonomy from an average of 3.1 (SD = 0.7) in the 2 years before the recruitment (T3) to an average of 3.7 (SD = 0.2) at the recruitment time (T1).

During the two years observation, the variations in the SE membership status of the 33 SE participants were assessed:

i) 9 (27.3%) employee members maintained a temporary contract.
ii) 11 (33.3%) employee members acquired a permanent contract;
iii) 5 (15.2%) employee members maintained the permanent contract throughout the two-years
iv) one person (3.0%) progressed from the role of trainee to employee member;
v) 7 (21.2%) trainees kept being trainees;

Supplementary Table 2 shows that over the two years, all SE members with SMI, on the one hand, set increasing numbers of objectives in all three domains, and these reflected greater confidence, skills and autonomy, and, on the other, needed a reschedule of the workload and job activities because of a decline in performance (probably because of the presentation of mental health crisis or progressive functioning deterioration due to the natural course of the mental disorder). The Supplementary Table 2 shows a progressive increase in requests from mental health professionals in terms of improving social skills.

### Satisfaction on SE job placement

Twenty-one SE participants (63.3%) provided ratings of their satisfaction with the job placement. High levels of satisfaction were reported with all aspects of the placement (6.0, SD = 0.2 on 7-point Likert scale) (see Table 2), with the highest scores for items concerning solidarity with colleagues and mental health professionals in coping with difficulties (6.3, SD = 0.9), and the feeling of playing an active part in the cooperative mission (6.3, SD = 1.2). The lowest scores were for items related to the working hours required (5.6, SD = 1.7).

**Table 2:** Satisfaction on the job placement in the SE at the recruitment time (T1 – year 2018); *n* = 21 subjects (63.6%). 7-point Likert scale (from 1 = the worst as possible to 7 = the best as possible). Data are described as Mean (SD) from the highest to lowest scores for the satisfaction on SE job placement

*In times of difficulty, can you count on the support of your colleagues at work? (20)*	6.3 (0.9)
*In times of difficulty, can you count on the support of mental health workers? (20)*	6.3 (0.9)
*Do you feel you contribute to the cooperative mission? (20)*	6.3 (1.2)
*How important is your work activity for a good quality of life?*	6.2 (1.1)
*Is the cooperative attentive and flexible towards your weaknesses and frailties?*	6.2 (1.1)
*How much do you value the work done by your colleagues? (20)*	6.2 (0.9)
*Are you satisfied with the tasks you do?*	6.1 (1.4)
*Does the balance in terms of work and private life satisfy you? (20)*	6.1 (1.2)
*Does your opinion mean in the cooperative? (20)*	6.0 (1.0)
*Do you feel an integral part of the cooperative?*	5.9 (1.4)
*Are you satisfied with professional relationships created in the Cooperative? (20)*	5.8 (1.4)
*Do you feel valued in the workplace?*	5.7 (1.6)
*Does the cooperative make it easier for you to express best your skills, abilities, and potential?*	5.7 (1.6)
*How satisfied are you of the number of hours to achieve your required tasks? (20)*	5.6 (1.7)
*Total*	**6.0 (0.2)**

### SE participants’ functioning over time

SE participants showed a higher significant improvement in functioning (t = -5.013, df = 32, *p* < 0.001) at the recruitment time (T1) than when they joined the SE (T0)—when they had been employed for an average of 5 years. It is remarkable that the GAF score increased of 10 points from when they joined the co-op (mean GAF score increased from 50.3 (SD = 11.0) when SE users joined the co-op (T0) to 60.5 (SD = 13.6) at the recruitment time (T1).

### The cross-sectional study comparing SE members with a matched group of people with SMI unemployed and not members of the SE

The matched comparison group was drawn from the participants of the VALERE-REC Study (eVALuation of outcomE in Residential—use of clinical data with REsearch objeCtives) [33, 40]. It was a survey that, developed from 2014 to 2015, involved 167 patients from 25 out of the 30 mental health supported accommodations in Verona, and aimed to evaluate clinical, social, and rehabilitative outcomes.

One to one matching of individuals in the SE group and the comparison group was based on primary diagnosis and years of contact with mental health services. Exact matching for primary diagnosis was possible in 97% of cases. Years of contact with services were categorized into bands (1–5, 6–10, 11–20, 21–30, 31 + years) and exact matching was achieved in 78% of cases (adjacent year categories were used in 15.6% of cases) (see Table 3).

**Table 3:** Comparison of SE members at the recruitment time (T1 – year 2018) and the comparison group on sociodemographic and clinical characteristics, functioning (GAF), symptoms (BPRS), and quality of life (MANSA)

	**Social cooperative** ***N* = 33**	**Supported accommodation** ***N* = 33**	***p*-value**
**Sociodemographic characteristics**			
Mean (SD) age in years	46.1 (11.3)	47.6 (11.7)	0.565
Male, N (%)	15 (45.5%)	17 (51.5%)	0.622
Marital status, N (%) (*n* = 32 for each group)			
No current partner	19 (59.4%)	26 (81.3%)	
Married/in partnership	3 (9.4%)	2 (6.2%)	0.145
Divorced/widowed	10 (31.2%)	4 (12.5%)	
Current living situation (*n* = 29 for each group)			
Supported accommodation	14 (48.3%)	33 (100%)	**-**
Independent living (with partner or relatives)	15 (51.7%)	0 (0%)	
**Primary psychiatric diagnosis (ICD-10), N (%)**			
Schizophrenia (F20)	7 (21.2%)	7 (21.2%)	
Non-affective non-schizophrenic diagnosis (F21-F24; F26-F29)	5 (15.2%)	5 (15.2%)	0.997
Affective psychosis (F25, F30.2, F31.2, F31.5, F31.6, F32.3)			
Other mood disorders (F30-39)	5 (15.2%)	6 (18.2%)	
Other disorders (e.g. anxiety, personality disorders) (F40-F48, F60-F69, F80-F99)	8 (24.2%) 8 (24.2%)	8 (24.2%) 7 (21.2%)	
**Problematic substance use, N (%) (*n* = 29 for each group)**	2 (6.1%)	2 (6.1%)	-
**At least one physical health comorbidity, N (%) (*n* = 24 for each group)**	12 (50.0%)	10 (41.7%)	0.562
**Contact with mental health services (years), Mean (SD)**	15.9 (9.5) 1 missing	16.0 (8.8)	0.953
**At least one previous admission to the acute psychiartric unit (*n* = 32 for each group)**	28 (87.5%)	29 (90.6%)	-
**In receipt of invalidity pension, N (%)**	24 (72.7%)	31 (93.9%)	-
**Functioning – Mean (SD) GAF score (max = 100)**	60.5 (13.7)	46.0 (17.2)	**0.001**
**Symptoms—Mean (SD) BPRS score**			
**(1 = no symptom; 7 = very severe symptom)**			
Depression/anxiety	1.8 (0.6)	2.5 (1.0)	**0.031**
Negative symptoms	1.6 (0.6)	2.8 (1.6)	**0.006**
Positive symptoms	1.5 (0.4)	2.3 (1.3)	**0.016**
Manic excitement	1.5 (0.7)	2.3 (1.6)	**0.028**
Cognitive symptoms	1.3 (0.5)	2.0 (1.1)	**0.020**
Total	1.6 (0.3)	2.4 (1.0)	**0.007**
**Quality of life—Mean (SD) MANSA** **(1 = not at all satisfied; 7 = extremely satisfied)** ***Main items***			
Being employed/unemployed/retired (*n* = 28 for each group)	5.4 (1.8)	4.3 (1.6)	**0.003**
Sexual life	3.9 (2.1)	2.6 (0.99)	**0.002**
Have a close friend (*n* = 29 for each group), N (%)	23 (79.3%)	16 (55.2%)	**0.050**
***Dimensions***			
Living	5.1 (1.4)	5.2 (1.0)	0.650
Health	4.6 (1.6)	4.6 (1.1)	0.258
Total	4.8 (1.1)	4,8 (0.8)	**0.034**

As shown in Table 3 there were no statistically significant differences between SE member participants and matched compared group in sociodemographic characteristics (n.s.), or substance misuse (n.s.). However, there was statistically significant difference between the two groups in ratings of global functioning (SE group mean GAF 60.5 (SD 13.7), comparison group mean GAF 46.0 (SD 17.2), t = 3.661, df = 32, *p* = 0.001). SE members had lower symptom severity scores than the comparison group (BPRS, t = -3.070, df = 18, *p* = 0.007), especially in terms of negative symptoms (t = -2.656, df = 18, *p* = 0.006). SE members also rated their overall QoL significantly higher than the comparison group (MANSA, t = 3.549, df = 29, *p* = 0.034) and rated greater satisfaction in various aspects of their life, including work situation (t = 3.428, df = 27, *p* = 0.002), and their social (t = 3.326, df = 29, *p* = 0.050) and sexual (t = 3.417, df = 29, *p* = 0.002) relationships (see Table 3).

## Discussion

The purpose of this study was to evaluate if a SE that uses recovery-oriented programs of supported employment for people with SMI improved psychosocial and work outcomes and favoured the achievement of personal recovery. We found that this approach helped a large proportion of members to achieve personal, social, and work-related goals and to gain skills, confidence and autonomy.

SE participants presented, over time, ratings of global function improved and had higher functioning, less severe symptoms and greater quality of life than the matched compared users with SMI.

These findings confirm literature that shows that QoL is higher in people who play a significant role in adult life than in those who do not [13, 41, 42].

The job maintenance of this sample with SMI was comparable with other data in literature [28]. The SE productivity was always guaranteed in the competitive market with a continuous performance of services provided to the private clients (e.g. hotels, restaurants, supermarkets, gardens). These results were achieved thanks to the continuous mental health professionals’ effort to personalize job placement, which in the SE setting represents a fundamental ingredient to maintain and achieve work outcomes. This flexibility permitted to users to maintain the job and reach a career growth.

Job performance improvement of members with a disability was also guaranteed by the holistic approach of mental health professionals. As already observed in other studies [10, 11, 27, 31], through working in a social enterprise people may implement not only work skills but also psychosocial ones. For example, the socializing activities proposed by the cooperative have the potential to act not only on group communication skills, so to create a good atmosphere and a reduction of conflict and burn-out [43] but also on individual social skills.

SE participants described high levels of satisfaction, and experienced social enterprises as an environment that promotes a restored sense of community, feelings of belonging, success, and competence thanks to the flexible and supportive atmosphere of the cooperative [31].

The findings regarding greater autonomy over time and functioning than the compared group are also encouraging, suggesting that the cooperative model enables people to become more independent and empowered. This may lead to individuals requiring less support to manage their mental health problems and daily activities, and it appears that the approach can play an important role in supporting recovery for people with SMI [44, 45]. Ultimately, the work in the SE may get access not only to rewarding job opportunities in the labour market but also to the reduction of the perception of being discriminated and stigmatized [27].

## Strengths and limitations

The results of the study need to be weighed against some limitations.

We are aware that we were limited by the relatively small sample size which may have biased our results. So this study is fundamentally a pilot one. Thus, it would be useful to conduct studies involving multiple SEs to ensure an adequate sample size to satisfy statistical power for data analysis. However, data about trainees and employee members, that, as expected, reported differences in autonomies, functioning, symptoms severuty and QoL in favour of the second category, suggests that data are coherent with the reality of the SE described.

Second, although the comparison study allowed to compare a group of people with similar types of SMI and lengths of contact with services, we cannot assume that the differences we found between the two groups are attributable to the cooperative. For example, it is possible that people with lower symptom severity were simply abler to engage in work related activities. In other words, our findings do not allow any inference about causality and further studies, ideally using randomised controlled deigns are warranted to evaluate the efficacy of the cooperative model.

Third, our ratings of participants’ personal, social and work objectives and autonomy were based on case records and may have been subject to observer/rater bias.

Fourth, a minor limitation is that the two matched samples were only partially comparable considering that only half SE members lived in a supported accommodation than the comparison group compounded by users all living in supported accommodation. The comparison group might include patients with more severe psychopathological symptoms and a poorer functioning that deeply affect the conduct of a normal adult life, so to need of more intense rehabilitative interventions [46, 47].

## Conclusions

In this paper, we presented a pilot study of an Italian model of job placement for people with SMI carried on by a B-type social cooperative with a ‘special ingredient’.

Our findings suggest that SEs that provide tailored support to assist people to gain skills and confidence may be an effective component in helping people in their recovery from SMI.

Even though larger and more robust studies are needed, this promising preliminary analysis represents a starting point to better understand the Italian model of supported employment based on SE.

## Supplementary Information

**Additional file 1.**

## Data Availability

The datasets used and/or analysed during the current study available from the corresponding author on reasonable request.

## Abbreviations

BPRS: Brief Psychiatric Rating Scale
co-ops: Cooperatives
GAF: Global Assessment of Functioning
IPS: Individual Placement Support
MANSA: Manchester Short Assessment of Quality of Life
PCR: Psychiatric Case Register
QoL: Quality of life
SE: Social enterprise
SMI: Severe mental illness
VALERE-REC: EVALuation of Outcome in Residential – use of clinical data with Research objeCtives
