# Supplementary material for: Supported employment for people with severe mental illness: a pilot study of an Italian social enterprise with a special ingredient
Source: BMC Psychiatry. 2022 Apr 26;22:296. doi: 10.1186/s12888-022-03881-8 (PMC9040313; doi:10.1186/s12888-022-03881-8)
Supplement: Supplementary file 1 — Additional file 1. [file 12888_2022_3881_MOESM1_ESM.docx]

**Supplementary Table 1 SE users’ personal, social and work related autonomy evaluated by mental health professionals 2 years before the recruitment time (T3), 1 year before the recruitment time (T2) and at the recruitment time (T1). Users were divided in two groups according to their SE membership status at the recruitment time (T1): 7 (21.2%) trainees, and 26 (78.8%) employees. Each participant’s personal, social and work autonomy was rated using a 5-point Likert scale: from 1 = very poor autonomy to 5 = excellent autonomy. Each graphic shows mean Likert scores for each group, meand (SD).**

|  | **2 years before the recruitment (T3)** | **1 year before the recruitment (T2)** | **Recruitment date (T1)** |
| --- | --- | --- | --- |
| **Personal autonomy** | | | |
| *Trainees, mean (SD)* | - | 2.0 (0.0)  (3 users) | 2.0 (0.0)  (5 users) |
| *Employees, mean (SD)* | 3.4 (0.5)  (7 users) | 3.5 (1.0)  (22 users) | 3.9 (0.9) |
| *Total, mean (SD)* | 3.4 (0.5)  (7 users) | 3.4 (1.1)  (25 users) | 3.6 (1.1)  (31 users) |
| **Social autonomy** | | | |
| *Trainees, mean (SD)* | - | 1.8 (0.5)  (3 users) | 1.8 (0.4)  (5 users) |
| *Employees, mean (SD)* | 2.9 (0.7)  (7 users) | 3.3 (0.9)  (22 users) | 3.7 (0.8) |
| *Total, mean (SD)* | 2.9 (0.7)  (7 users) | 3.2 (0.9)  (25 users) | 3.4 (1.1)  (31 users) |
| **Work autonomy** | | | |
| *Trainees, mean (SD)* | - | 2.0 (0.0)  (3 users) | 2.0 (0.0)  (5 users) |
| *Employees, mean (SD)* | 3.0 (1.0)  (7 users) | 3.3 (0.9)  (22 users) | 3.6 (1.0) |
| *Total, mean (SD)* | 3.0 (1.0)  (7 users) | 3.2 (1.0)  (25 users) | 3.3 (1.1)  (31 users) |
| 5-point Likert scale: from 1 = very poor autonomy to 5 = excellent autonomy | | | |

**Supplementary Table 2 Distribution of SE members’ personal, social and work related objectives proposed by mental health professionals 2 years before the recruitment time (T3), 1 year before the recruitment time (T2) and at the recruitment time (T1).**

|  | **2 years before the recruitment (T3)**  **7 users** | **1 year before the recruitment (T2)**  **24 users** | **Recruitment date (T1)**  **33 users** |
| --- | --- | --- | --- |
| **Personal related objectives** | | | |
| *Maintenance of the status quo, N (%)* | 3 (42.9%) | 16 (66.7%) | 14 (42.4%) |
| *Improvement of mental health management, N (%)* | 1 (14.3%) | 6 (25.1%) | 11 (36.4%) |
| *Improvement of daily life skills, N (%)* | 1 (14.3%) | 3 (12.6%) | 4 (12.0%) |
| *Improvement of daily life activities management, N (%)* | 1 (14.3%) | 2 (8.4%) | 4 (12.0%) |
| *Move to an independent living, N (%)* | 0 (0.0%) | 0 (0.0%) | 2 (6.1%) |
| *Monitoring of personal autonomy, N (%)* | 1 (14.3%) | 2 (8.4%) | 4 (12%) |
| **Social related objectives** | | | |
| *Maintenance of the status quo, N (%)* | 1 (14.3%) | 6 (26.1%)  (23 users) | 8 (24.2%) |
| *Improvement of social skills, N (%)* | 4 (57.1%) | 12 (52.2%)  (23 users) | 21 (63.0%) |
| *Increase of assertiveness, N (%)* | 0 (0.0%) | 2 (8.7%)  (23 users) | 4 (12.0%) |
| *Increase relationships inside the SE, N (%)* | 4 (57.1%) | 6 (26.1%)  (23 users) | 8 (24.2%) |
| *Increase relationships outside the SE, N (%)* | 1 (14.3%) | 2 (8.7%)  (23 users) | 1 (3.0%) |
| *Monitoring of social autonomy, N (%)* | 1 (14.3%) | 2 (8.7%)  (23 users) | 2 (6.1%) |
| **Work related objectives** | | | |
| *Maintenance of the status quo, N (%)* | 1 (14.3%) | 6 (25.0%) | 7 (21.2%) |
| *Reducing load or working hours/week, N (%)* | 2 (28.6%) | 0 (0.0%) | 4 (12.0%) |
| *Acquiring new skills, N (%)* | 0 (0.0%) | 3 (12.4%) | 2 (6.1%) |
| *To develop or strengthen work skills, N (%)* | 2 (28.6%) | 15 (62.5%) | 6 (18.2%) |
| *Gaining greater work autonomy, N (%)* | 0 (0.0%) | 3 (12.5%) | 2 (6.1%) |
| *Improving soft skills, N (%)* | 5 (71.5%) | 7 (29.2%) | 22 (66.0%) |
| *Working on motivation, N (%)* | 0 (0.0%) | 0 (0.0%) | 3 (9.0%) |
| *Monitoring work autonomy and ability to perform a task, N (%)* | 0 (0.0%) | 0 (0.0%) | 0 (0%) |
